# Correction: Global analysis of cell behavior and protein dynamics reveals region-specific roles for Shroom3 and N-cadherin during neural tube closure

**DOI:** 10.7554/eLife.100778

**Published:** 2024-07-02

**Authors:** Austin T Baldwin, Juliana Kim, Hyemin Seo, John B Wallingford

**Keywords:** *Xenopus*

Baldwin AT, Kim JH, Seo H, Wallingford JB. 2022. Global analysis of cell behavior and protein dynamics reveals region-specific roles for Shroom3 and N-cadherin during neural tube closure. *eLife* **11**:e66704. doi: 10.7554/eLife.66704.

Published 4 March 2022

A colleague has informed us that the graph in the lower right panel of Figure 5C of this paper is a duplicate of the graph in the upper right of the same figure panel. We regret this error, and the correct graph has been added in this correction. Crucially, this change has no impact on the conclusions of the paper, as the text was written referring to a previous iteration of this figure with the correct graph. Thus, no changes to the main text were made relating to this error.

The corrected Figure 5 is shown here:

**Figure 1:**
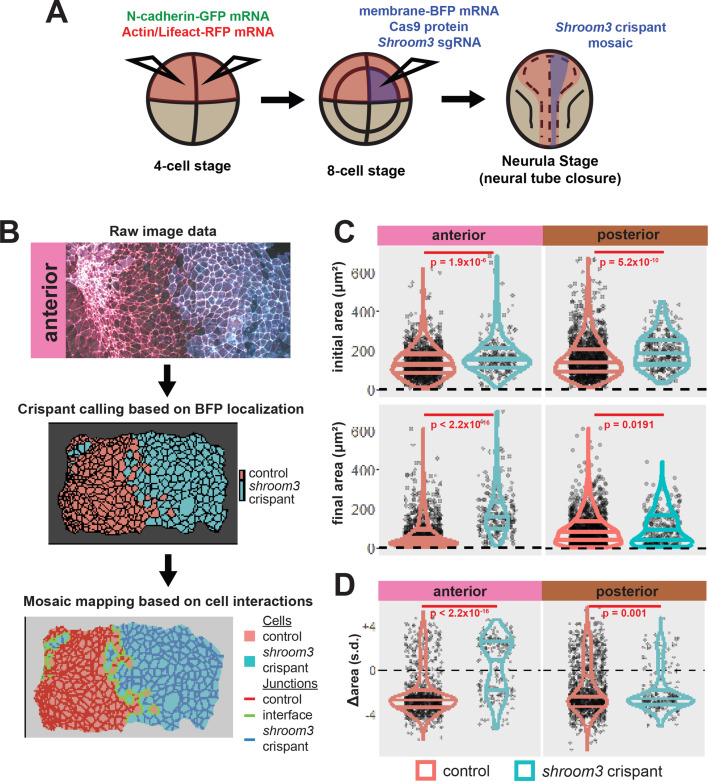


The originally published Figure 5 is shown for reference:

**Figure 2:**
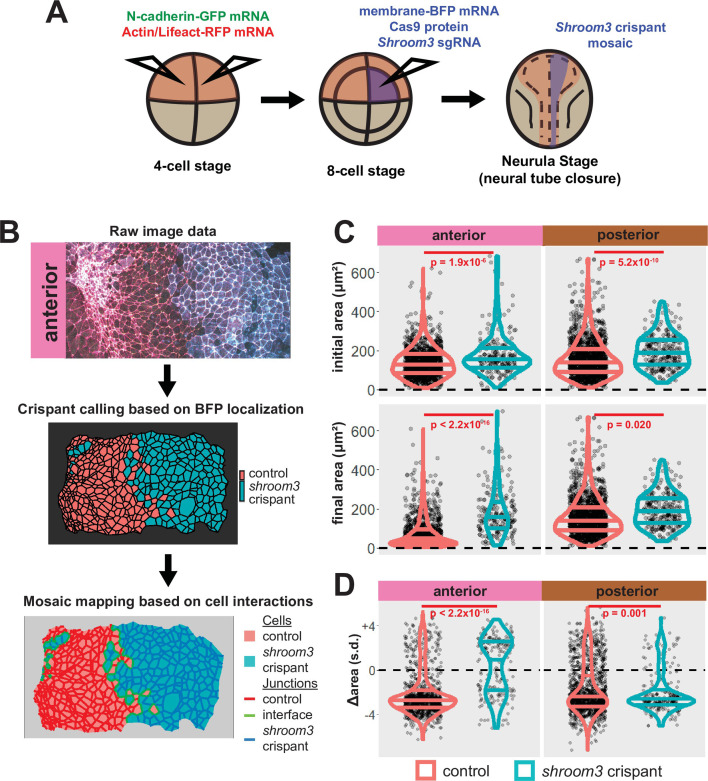


Additionally, when we carefully examined the manuscript in light of the duplicated figure, we found that several of the figure legends were inaccurate, as they described previous iterations of the figures. The corrections to the figure legends are as follows.

The corrected Figure 6 legend:

Figure 6: Loss of *shroom3* disrupts actin and N-cadherin accumulation and constriction in the anterior neural ectoderm. (A) Representative images of LifeAct/actin and N-cadherin-GFP (N-cad-GFP) localization in control cells (left) and *shroom3* crispant cells (right) from the anterior region of the neural ectoderm. Scale bar = 15 µm. (B) Distribution of overall change (Δ) in medial LifeAct/actin (standardized) from anterior cells. (C) Distribution of overall change (Δ) in junctional LifeAct/actin (standardized) from anterior cells. (D) Distribution of overall change (Δ) in medial N-cadherin (standardized) from anterior cells. (E) Distribution of overall change (Δ) in junctional N-cadherin-GFP (standardized) from anterior cells. In B-E, horizontal lines on density plots/violins indicate quartiles of distribution, black circles are individual cells, and statistical comparisons performed by KS test.

The originally published Figure 6 legend is shown for reference:

Figure 6. Medial actin accumulation drives apical constriction while loss of *shroom3* disrupts actin accumulation and constriction in the anterior neural ectoderm. (A) Representative images of LifeAct/actin localization in control cells (left) and *shroom3* crispant cells (right) from the anterior region of the neural ectoderm. Scale bar = 15 µm. (B) Distribution of overall change (Δ) in medial LifeAct/actin (standardized) from anterior cells. (C) Distribution of overall change (Δ) in junctional LifeAct/actin (standardized) from anterior cells. In B and C, horizontal lines on density plots/violins indicate quartiles of distribution, black circles are individual cells, and statistical comparisons performed by Kolmogorov-Smirnov (KS) test. (D and E) 2D distribution of changes in apical area and medial (D) or junctional (E) LifeAct/actin (both standardized). Percentages in white indicate the percentage of total cells in each quadrant. Statistical comparisons performed by Peacock test, a 2D implementation of the KS test. (F and G) 2D density plots of all observations of apical area versus medial (F) or junctional (G) LifeAct/actin for all cells within each group. Red lines indicate best-fit line through the observations. Statistics (r and p) are calculated for Pearson’s correlation. Cells situated along the mosaic interface were excluded from these analyses. s.d.=standard deviation.

The corrected Figure 7 legend:

Figure 7: Medial N-cadherin accumulation is severely disrupted in anterior *shroom3* crispant cells that fail to apically constrict. (A–D) 2D density plots of all observations of apical area versus medial (A) or junctional (B) LifeAct/actin or medial (C) or junctional (D) N-cadherin for all cells within each group. Percentages in white indicate the percentage of total cells in each quadrant. Statistical comparisons performed by Peacock test, a 2D implementation of the KS test. Cells situated along the mosaic interface were excluded from these analyses. s.d.=standard deviation.

The originally published Figure 7 legend is shown for reference:

Figure 7. Medial N-cadherin accumulation is severely disrupted in anterior *shroom3* crispant cells that fail to apically constrict. (A) Representative images of N-cadherin localization in control cells (left) and *shroom3* crispant cells (right) from the anterior region of the neural ectoderm. Scale bar = 15 µm. (B) Distribution of overall change (Δ) in medial N-cadherin (standardized) from anterior cells. (C) Distribution of overall change (Δ) in junctional N-cadherin (standardized) from anterior cells. In B and C, horizontal lines on density plots/violins indicate quartiles of distribution, black circles are individual cells, and statistical comparisons performed by Kolmogorov-Smirnov (KS) test. (D and E) 2D distribution of changes in apical area and medial (D) or junctional (E) N-cadherin (both standardized). Percentages in white indicate the percentage of total cells in each quadrant. Statistical comparisons performed by Peacock test, a 2D implementation of the KS test. (F and G) 2D density plots of all observations of apical area versus medial (F) or junctional (G) N-cadherin for all cells within each group. Red lines indicate best-fit line through the observations. Statistics (r and p) are calculated for Pearson’s correlation. Cells situated along the mosaic interface were excluded from these analyses. s.d.=standard deviation.

The corrected Figure 8 legend:

Figure 8: Actin and N-cadherin accumulation are uncoupled in anterior *shroom3* crispant cells. (A and B) 2D density plots of all observations of medial (A) or junctional (B) LifeAct/actin versus apical area for all cells within each group. (C and D) 2D density plots of all observations of medial (C) or junctional (D) LifeAct/actin versus N-cadherin at the same domain for all cells within each group. Red lines indicate best-fit line through the observations. Statistics (r and p) are calculated for Pearson’s correlation. Cells situated along the mosaic interface were excluded from these analyses. s.d.=standard deviation.

The originally published Figure 8 legend is shown for reference:

Figure 8. Actin and N-cadherin accumulation are uncoupled in anterior *shroom3* crispant cells. (A and B) 2D density plots of all observations of medial (A) or junctional (B) LifeAct/actin versus N-cadherin for all cells within each group. Red lines indicate best-fit line through the observations. Statistics (r and p) are calculated for Pearson’s correlation. Cells situated along the mosaic interface were excluded from these analyses. s.d.=standard deviation.

The corrected Figure 9 legend:

Figure 9: Loss of *shroom3* disrupts actin dynamics in the posterior neural ectoderm. (A) Representative images of LifeAct/actin and N-cadherin-GFP (N-cad-GFP) localization in control cells (left) and *shroom3* crispant cells (right) from the posterior region of the neural ectoderm. White asterisks mark the same cell in each embryo. Scale bar = 15 µm. (B) Distribution of overall change (Δ) in medial LifeAct/actin (standardized) from anterior cells. (C) Distribution of overall change (Δ) in junctional LifeAct/actin (standardized) from anterior cells. (D) Distribution of overall change (Δ) in medial N-cadherin (standardized) from anterior cells. (E) Distribution of overall change (Δ) in junctional N-cadherin-GFP (standardized) from anterior cells. In B-E, horizontal lines on density plots/violins indicate quartiles of distribution, black circles are individual cells, and statistical comparisons performed by KS test.

The originally published Figure 9 legend is shown for reference:

Figure 9. Loss of *shroom3* disrupts actin dynamics in the posterior neural ectoderm. (A) Representative images of LifeAct/actin localization in control cells (left) and *shroom3* crispant cells (right) from the posterior region of the neural ectoderm. White asterisks mark the same cell in each embryo. Scale bar = 15 µm. (B) Distribution of overall change (Δ) in medial LifeAct/actin (standardized) from posterior cells. (C) Distribution of overall change (Δ) in junctional LifeAct/actin (standardized) from anterior cells. In (B and C), horizontal lines on density plots/violins indicate quartiles of distribution, black circles are individual cells, and statistical comparisons performed by Kolmogorov-Smirnov (KS) test. (D and E) 2D distribution of changes in apical area and medial (D) or junctional (E) LifeAct/actin (both standardized). Percentages in white indicate the percentage of total cells in each quadrant. Statistical comparisons performed by Peacock test, a 2D implementation of the KS test. (F and G) 2D density plots of all observations of apical area versus medial (F) or junctional (G) LifeAct/actin for all cells within each group. Red lines indicate best-fit line through the observations. Statistics (r and p) are calculated for Pearson’s correlation. Cells situated along the mosaic interface were excluded from these analyses. s.d.=standard deviation.

The corrected Figure 10 legend:

Figure 10: Actin and N-cadherin dynamics are highly heterogenous in the posterior neural ectoderm and poorly correlated with apical constriction. (A and B) 2D density plots of all observations of apical area versus medial (A) or junctional (B) LifeAct/actin for all cells within each group. (C and D) 2D density plots of all observations of apical area versus medial (C) or junctional (D) N-cadherin for all cells within each group. Red lines indicate best-fit line through the observations. Statistics (r and p) are calculated for Pearson’s correlation. Cells situated along the mosaic interface were excluded from these analyses. s.d.=standard deviation.

The originally published Figure 10 legend is shown for reference:

Figure 10. N-cadherin dynamics are highly heterogenous in the posterior neural ectoderm and poorly correlated with apical constriction. (A) Representative images of N-cadherin localization in control cells (left) and *shroom3* crispant cells (right) from the posterior region of the neural ectoderm. White asterisks mark the same cell in each embryo. Scale bar = 15 µm. (B) Distribution of overall change (Δ) in medial N-cadherin (standardized) from posterior cells. (C) Distribution of overall change (Δ) in junctional N-cadherin (standardized) from posterior cells. In (B and C), horizontal lines on density plots/violins indicate quartiles of distribution, black circles are individual cells, and statistical comparisons performed by Kolmogorov-Smirnov (KS) test. (D and E) 2D distribution of changes in apical area and medial (D) or junctional (E) N-cadherin (both standardized). Percentages in white indicate the percentage of total cells in each quadrant. Statistical comparisons performed by Peacock test, a 2D implementation of the KS test. (F and G) 2D density plots of all observations of apical area versus medial (F) or junctional (G) N-cadherin for all cells within each group. Red lines indicate best-fit line through the observations. Statistics (r and p) are calculated for Pearson’s correlation. Cells situated along the mosaic interface were excluded from these analyses. s.d.=standard deviation.

The corrected Figure 11 legend:

Figure 11: Individual junction behaviors are polarized in the posterior neural ectoderm. (A) Junction orientation from posterior control embryo from Figure 2. Scale bars = 100 µm. (B) Distribution of overall change (Δ) in junction length (standardized) from the posterior neural ectoderm. Horizontal lines on density plots/violins indicate quartiles of distribution, black circles are individual cells, and statistical comparisons performed by Kolmogorov-Smirnov (KS) test. (C) 2D density plots of all observations of mean junction orientation over time versus overall change (Δ) in junction length (standardized) for all junctions within each group. Dashed cyan ellipses indicate areas of altered polarization between control and shroom3 crispant junctions. (D) 2D density plots of all observations of mean junction orientation over time versus overall change (Δ) in junction actin (standardized) for all junctions within each group. (E) 2D density plots of all observations of mean junction orientation over time versus overall change (Δ) in junction N-cadherin (standardized) for all junctions within each group. Percentages in white indicate the percentage of total cells in each quadrant. Statistical comparisons performed by Peacock test, a 2D implementation of the KS test. s.d. = standard deviation. (F) Example diagram of T1 transition/neighbor exchange within an epithelial tissue. (G) Distribution of stable T1 transitions/neighbor exchanges per cell as calculated by Tissue Analyzer. Horizontal lines on density plots/violins indicate quartiles of distribution, black circles are individual cells, and statistical comparisons performed by KS test. (H) Histogram showing relative frequencies of mean junction orientation from shrinking (Δ length < 0, upper panels) and growing (Δ length > 0, lower panels) posterior junctions. Compare to 11C. Statistical comparisons performed by KS test.

The originally published Figure 11 legend is shown for reference:

Figure 11: Individual junction behaviors are polarized in the posterior neural ectoderm. (A) Distribution of overall change (Δ) in junction length (standardized) from the posterior neural ectoderm. Horizontal lines on density plots/violins indicate quartiles of distribution, black circles are individual cells, and statistical comparisons performed by Kolmogorov-Smirnov (KS) test. (B) Junction orientation from posterior control embryo from Figure 2. Scale bars = 100 µm. (C) 2D density plots of all observations of mean junction orientation over time versus overall change (Δ) in junction length (standardized) for all junctions within each group. Dashed cyan ellipses indicate areas of altered polarization between control and shroom3 crispant junctions. (D) 2D density plots of all observations of mean junction orientation over time versus overall change (Δ) in junction actin (standardized) for all junctions within each group. (E) 2D density plots of all observations of mean junction orientation over time versus overall change (Δ) in junction N-cadherin (standardized) for all junctions within each group. Percentages in white indicate the percentage of total cells in each quadrant. Statistical comparisons performed by Peacock test, a 2D implementation of the KS test. s.d. = standard deviation.

The corrected Figure 11—figure supplement 1 legend:

Figure 11—figure supplement 1: Individual junction behaviors are mainly anisotropic in the neural ectoderm. (A) Distribution of overall change (Δ) in junction length (standardized) from the anterior neural ectoderm. Horizontal lines on density plots/violins indicate quartiles of distribution, black circles are individual cells, and statistical comparisons performed by Kolmogorov-Smirnov (KS) test. (B) 2D density plots of all observations of mean junction orientation over time versus overall change (Δ) in junction length (standardized) for all junctions within each group. (C) 2D density plots of all observations of mean junction orientation over time versus overall change (Δ) in junction actin (standardized) for all junctions within each group. (D) 2D density plots of all observations of mean junction orientation over time versus overall change (Δ) in junction N-cadherin (standardized) for all junctions within each group. Percentages in white indicate the percentage of total cells in each quadrant. Statistical comparisons performed by Peacock test, a 2D implementation of the KS test. s.d. = standard deviation.

The originally published Figure 11—figure supplement 1 legend is shown for reference:

Figure 11—figure supplement 1: Individual junction behaviors are mainly anisotropic in the neural ectoderm. (A) Distribution of overall change (Δ) in junction length (standardized) from the anterior neural ectoderm. Horizontal lines on density plots/violins indicate quartiles of distribution, black circles are individual cells, and statistical comparisons performed by Kolmogorov-Smirnov (KS) test. (C) 2D density plots of all observations of mean junction orientation over time versus overall change (Δ) in junction length (standardized) for all junctions within each group. (D) 2D density plots of all observations of mean junction orientation over time versus overall change (Δ) in junction actin (standardized) for all junctions within each group. (E) 2D density plots of all observations of mean junction orientation over time versus overall change (Δ) in junction N-cadherin (standardized) for all junctions within each group. Percentages in white indicate the percentage of total cells in each quadrant. Statistical comparisons performed by Peacock test, a 2D implementation of the KS test. s.d. = standard deviation.

